# High genetic diversity and different type VI secretion systems in *Enterobacter* species revealed by comparative genomics analysis

**DOI:** 10.1186/s12866-023-03164-6

**Published:** 2024-01-19

**Authors:** Mu Peng, Weiyuan Lin, Aifen Zhou, Zhihui Jiang, Fangzhen Zhou, Zhiyong Wang

**Affiliations:** 1 Hubei Key Laboratory of Biological Resources Protection and Utilization, Hubei Minzu University, Enshi, China; 2 College of Biological and Food Engineering, College of Biological and Food Engineering, Hubei Minzu University, Hubei Minzu University, No. 39 Xueyuan Street, Enshi, 445000 China; 3 Institute for Environmental Genomics, Department of Microbiology and Plant Biology, University of Oklahoma, Norman, OK USA

**Keywords:** *Enterobacter*, Comparative genomics, Pan-genomics, Type VI secretion system

## Abstract

The human-pathogenic *Enterobacter* species are widely distributed in diverse environmental conditions, however, the understanding of the virulence factors and genetic variations within the genus is very limited. In this study, we performed comparative genomics analysis of 49 strains originated from diverse niches and belonged to eight *Enterobacter* species, in order to further understand the mechanism of adaption to the environment in *Enterobacter*. The results showed that they had an open pan-genome and high genomic diversity which allowed adaptation to distinctive ecological niches. We found the number of secretion systems was the highest among various virulence factors in these *Enterobacter* strains. Three types of T6SS gene clusters including T6SS-A, T6SS-B and T6SS-C were detected in most *Enterobacter* strains. T6SS-A and T6SS-B shared 13 specific core genes, but they had different gene structures, suggesting they probably have different biological functions. Notably, T6SS-C was restricted to *E. cancerogenus*. We detected a T6SS gene cluster, highly similar to T6SS-C (91.2%), in the remote related *Citrobacter rodenitum*, suggesting that this unique gene cluster was probably acquired by horizontal gene transfer. The genomes of *Enterobacter* strains possess high genetic diversity, limited number of conserved core genes, and multiple copies of T6SS gene clusters with differentiated structures, suggesting that the origins of T6SS were not by duplication instead by independent acquisition. These findings provide valuable information for better understanding of the functional features of *Enterobacter* species and their evolutionary relationships.

**Supplementary Information:**

The online version contains supplementary material available at 10.1186/s12866-023-03164-6.

## Background

*Enterobacter* species are ubiquitous in nature and have been isolated from different ecological niches, such as plants, wastewater, human body and so on [1–5]. They exhibit considerable phenotypic and genomic diversities [6]. *Enterobacter* species are opportunistic pathogens and can cause infections in susceptible individuals, particularly those with compromised immune systems. Most studies on *Enterobacter* mainly focused on clinical diseases, such as nosocomial infection in humans, and suggested that it is a potential pathogenic bacteria [7], which can cause many kinds of human diseases [8]. Some *Enterobacter* strains show resistance to multiple antibiotics, posing a challenge for effective treatment [9]. Some *Enterobacter* strains are harmless commensals while some *Enterobacter* strains are potential pathogens, suggesting the complexity of *Enterobacter* species genomes and the need for further research to better understand their impact on human health. Protein secretion systems are critical for both pathogenic and nonpathogenic organisms to exploit nutrient resources within a host or specific niche and to evade recognition by the immune system [10, 11]. However, systematic analysis of protein secretion systems among *Enterobacter* species is very limited.

Several distinct protein secretion systems in bacterial pathogens are involved in invasion to their hosts [12]. So far, at least 6 types of secretion systems have been found in bacteria. Among these systems, Type VI secretion system (T6SS) has long been considered as a virulence factor of pathogenic bacteria. In addition to evading host immunity, T6SS is also associated with the competition for ecological niches and survival of bacteria under harmful environments [13]. Bacteria inject the effector proteins into the cells of intercellular competitors via T6SS to facilitate the inter-species bacterial competitions [14]. Therefore, T6SS represents crucial determinants of competitive fitness and pathogenic potential [15]. On the other hand, many beneficial bacteria utilize T6SS to promote plant growth by biofilm formation [16–18]. M Becker, S Patz, Y Becker, B Berger, M Drungowski, B Bunk, J Overmann, C Spröer, J Reetz and GV Tchuisseu Tchakounte [19] suggested that T6SS may provide endophytic bacteria advantages in environmental adaptability and host colonization. However, analysis of the distribution of T6SSs in *Enterobacter* species is very limited [20].

Comparative genomic analysis is a useful tool for exploring the evolution of species-specificity, it can provide evidence to better understand genomic rearrangement-related gene variations and deletions, horizontal gene transfer elements, and prophage-related sequence identification [21]. The increasing availability of whole genome sequences for bacteria enables the analysis of pan-genomes, encompassing core-, pan- and dispensable-genomes [22]. In addition, pan-genomics can be used for studying and modeling genomic diversity, evolution, adaptability, and population structure [23, 24]. Dispensable-genomes, which consists of genes shared by some but not all strains of a species, may also be involved in key activities of pathogenicity, drug resistance and stress response [23]. Although these dispensable genes may increase the adaptability of species to ecological niches, they are not necessary for their survival. Bacteria obtain some genes through horizontal gene transfer and these genes are often found in genome islands [25]. With the accumulation of bacterial genomes, comparative genomics is a better method to explore the phylogenetic relationship and the mechanism of environmental adaptation. To date, only few comparative genomics studies focusing exclusively on *E. cloacae* have been reported [20, 26–28]. There is no comparative genomics analysis involving multiple *Enterobacter* species. Here, we performed comparative analysis of eight *Enterobacter* species, including pan-genomics and the distribution of T6SS, in order to further understand the mechanism of adaption to the environment in *Enterobacter*.

## Methods

### Taxonomical evaluation by whole genome analysis

To ensure meaningful comparison between genomes and to analyze the genomic differences in different species, 49 representative *Enterobacter* strains belonging to eight *Enterobacter* species, each with complete genomes available in NCBI (https://www.ncbi.nlm.nih.gov/genome/), were selected based on completeness of genome sequences and diversity of habitats. The number of strains in each species ranged from 3 to 10. These strains were isolated from various habitats, including humans, plants, soils, wastewater, animals, and insects (Table 1). All genomes were uploaded and re-annotated on the Rapid Annotations using Subsystems Technology (RAST) server for SEED-based automated annotation and Clusters of orthologous Groups (COG) [29]. Basic Local Alignment Search Tool (BLAST) search was performed with all predicted open reading frames (ORFs) from the 49 strains against Virulence Factor Database (VFDB) [30] to identify potential genes encoding known virulence factors, respectively.

**Table 1:** Comparison of general features of genomes of 49 *Enterobacter* strains

Strain	Size (Mb)	GC content (%)	Gene number	Protein number	tRNA	rRNA	Source
*E. asburiae* 1808-013	4.77	55.90	4 587	4 402	71	25	Human
*E. asburiae* AEB30	4.75	55.80	4 522	4 363	83	25	Plant
*E. asburiae* ATCC 35953	4.81	55.43	4 738	4 405	83	25	Human
*E. asburiae* CAV1043	5.03	55.47	4 907	4 629	83	25	Human
*E. asburiae* ENIPBJ-CG1	4.65	55.80	4 490	3 835	84	25	Human
*E. asburiae* L1	4.56	56.10	4 354	4 213	84	25	Plant
*E. asburiae* MRY18-106	4.65	55.9	4 447	4 376	86	25	Human
*E. cancerogenus* CR-Eb1	4.80	55.80	4 576	4 371	85	25	Insect
*E. cancerogenus* JY65	4.74	55.77	4 715	4 497	82	13	Plant
*E. cancerogenus* MiY-F	4.99	55.54	4 680	4 528	83	25	Plant
*E. cloacae* E3442	4.72	55.53	4 643	4 392	76	11	Animal
*E. cloacae* GGT036	4.85	55.00	4 584	4 437	83	25	Soil
*E. cloacae* PIMB10EC27	5.42	54.38	5 390	4 964	85	25	Human
*E. cloacae* ATCC 13047	5.60	54.60	5 639	5 518	84	25	Human
*E. cloacae* SDM	4.97	55.10	4 736	4 521	80	25	Soil
*E. hormaechei* 34983	5.06	54.84	4 963	4 698	84	25	Human
*E. hormaechei* A1	5.38	54.96	5 262	4 951	84	25	Human
*E. hormaechei* C15	5.19	54.76	5 087	4 839	87	25	Human
*E. hormaechei* C126	5.03	54.95	5 443	995^a^	84	25	Human
*E. hormaechei* E5	5.22	54.79	5 136	4 828	85	25	Human
*E. hormaechei* L51	5.36	54.39	5 225	4 993	85	25	Human
*E. hormaechei* N1	5.11	55.23	5 038	4 716	87	25	Human
*E. hormaechei* MS7884A	5.27	54.87	5 194	4 960	82	22	Human
*E. hormaechei* DSM 16691	4.78	55.60	4 609	4 426	85	24	Human
*E. hormaechei* WCHEH090011	4.79	55.17	4 745	4 498	86	25	Human
*E. kobei* C16	5.37	54.43	5 351	5 075	86	25	Human
*E. kobei* EB_P8_L5_01.19	5.20	54.56	5 203	4 757	84	22	Human
*E. kobei* ENHKU01	4.73	55.10	4 551	4 327	83	25	Plant
*E. kobei* WCHEK045523	5.17	54.74	5 042	4 763	83	25	Human
*E. ludwigii* EcWSU1	4.80	54.53	4 671	4 460	84	25	Plant
*E. ludwigii* EN-119	4.95	54.60	4 689	4 511	86	28	Human
*E. ludwigii* I42	4.72	54.70	4 521	4 356	83	25	Soil
*E. ludwigii* JP9	4.68	54.80	4 451	4 177	83	25	Soil
*E. ludwigii* P101	5.37	54.40	5 289	5 134	100	25	Plant
*E. ludwigii* UW5	4.90	54.50	4 637	4 496	77	25	Soil
*E. roggenkampii* 704SK10	4.88	55.86	4 947	4 671	84	25	Wastewater
*E. roggenkampii* BP10374	4.85	56.10	4 719	4 292	84	25	Human
*E. roggenkampii* FDAARGOS_523	5.19	55.33	5 142	4 826	85	25	Human
*E. roggenkampii* R11	4.99	55.76	4 854	4 604	84	25	Wastewater
*E. roggenkampii* WCHER090065	4.95	55.83	4 791	4 544	82	25	Human
*Enterobacter* sp. 638	4.68	52.92	4 545	4 342	84	22	Plant
*Enterobacter* sp. Crenshaw	4.63	56.00	4 406	4 259	83	25	Plant
*Enterobacter* sp. E20	4.76	55.70	4 595	4 393	83	25	Soil
*Enterobacter* sp. EA-1	5.15	54.60	5 328	1 052^a^	81	22	Wastewater
*Enterobacter* sp. HK169	4.55	56.20	4 316	4 144	83	25	Plant
*Enterobacter* sp. N18-03635	4.69	55.60	4 530	4 335	87	25	Human
*Enterobacter* sp. ODB01	4.53	54.80	4 316	4 128	89	25	Soil
*Enterobacter* sp. R4-368	5.16	53.97	4 977	4 756	87	29	Plant
*Enterobacter* sp. SA187	4.43	56.00	4 149	4 004	84	22	Plant

^a^The number of pseudogenes in *Enterobacter hormaechei* C126 and *Enterobacter* sp. EA-1 is 4332 and 4165, respectively, as reported in the NCBI database (https://www.ncbi.nlm.nih.gov/nuccore/CP041054.1 and https://www.ncbi.nlm.nih.gov/nuccore/1329922488)

Efficient Database framework for comparative Genome Analyses using BLAST score Ratios (EDGAR) was used for core genome, pan genome and singleton analysis, average amino acid identity (AAI) and average nucleotide identity (ANI) [31]. The cutoff values of AAI and ANI for species identification are 95% [32]. In order to clarify the phylogenetic classification of *Enterobacter* spp., two phylogenetic trees were constructed according to concatenated amino acid sequences of core genes and house-keeping genes, using FastTree 2.1.8 with 1,000 bootstrap iterations [33].

### Pan- and core-genome analysis

The pan and core genome curves were performed by Bacterial Pan-Genome Analysis Pipeline (BPGA) [34]. The size of the pan-genome was speculated by a power law regression function *Ps* = κ*n*^γ^ using the BPGA pipeline. In this formula, if the exponent γ < 0, the pan-genome is closed and its size reaches a constant with addition of new sequencing genomes; if 0 < γ < 1, the pan-genome size is open and grows continuously [35].

### Identification of genes involved in bacterial type VI secretion system

The core component clusters of Bacterial Type VI Secretion System (T6SS) were predicted by VRprofile [36]. Thirteen T6SS core components were selected for further analysis: COG3501 (vgrG), COG0542 (clpG), COG3520 (impH), COG3519 (impC), COG3518 (impF), COG3517 (iglB), COG3157 (hcp1), COG3516 (iglA), COG3515 (impA), COG3523 (icmF), COG3455 (dotU), COG3522 (impJ) and COG3521 (vasD) [37]. T6SS clusters with less than thirteen core component genes were discarded. In general, the T6SS gene clusters are categorized in three families (i-iii) and the family i is divided into six subfamilies (1, 2, 3, 4a, 4b and 5) [38–41]. To identify the T6SS subfamilies in higher precision, 31 well-studied T6SSs were selected to rebuild the phylogenetic tree based on the amino acid sequence of TssC (impC) protein. The phylogenetic tree was constructed with MEGA6.0 using the neighbor-joining method with 1,000 bootstrap test [42].

## Results and discussion

### General features of *Enterobacter* genomes

The genomes of many *Enterobacter* strains have been sequenced in the past few years. These genome sequences data constitute an excellent resource for evaluating the genetic diversity and exploring the evolutionary relationship in *Enterobacter* genus. In this study, we analyzed high quality genome sequences of 49 strains that were isolated from different environments including 25 from human, 12 from plant, 7 from soil, 3 from wastewater, 1 from animal, and 1 from insect, and representing eight species (Table 1). An overview of the basic properties of *Enterobacter* genomes was presented in Table 1. The genome size ranged from 4.43 MB (*Enterobacter* sp. SA187) to 5.6 MB (*E. cloacae* ATCC 13047), the GC contents varied from 54.38% to 56.1%, and the numbers of genes per genome ranged from 4027 to 5639. Interestingly, strains C126 and EA-1 had the much less protein-encoding genes (995 and 1052, respectively) but more pseudo genes (4332 and 4165, respectively). The big variations among these genomes suggested that the evolution of *Enterobacter* is accompanied by genomic adaptation to their different hosts and environments.

### Genome sequence-based taxonomic classification of *Enterobacter* sp. strains

In this work, nine of the 49 strains were designated as *Enterobacter* sp. based on their 16S rRNA gene nucleic acid sequences, but were not assigned to a distinct species. To clarify the taxonomy of these strains, we calculated and compared the values of the average amino acid identity (AAI) and average nucleotide identity (ANI) of the 49 *Enterobacter* genomes. We found that *Enterobacter* sp. Crenshaw, *Enterobacter* sp. E20 and *Enterobacter* sp. HK169 showed identities of more than 98.8% with *Enterobacter asburiae*, while *Enterobacter* sp. ODB01 exhibited identities of 99.8% with *Enterobacter kobei* (Additional file 1)*.* Therefore, we suggest assigning strains Crenshaw, E20, HK169 and ODB01 as *E. asburiae* Crenshaw, *E. asburiae* E20, *E. asburiae* HK169 and *Enterobacter kobei* ODB01.

**Fig. 1:**
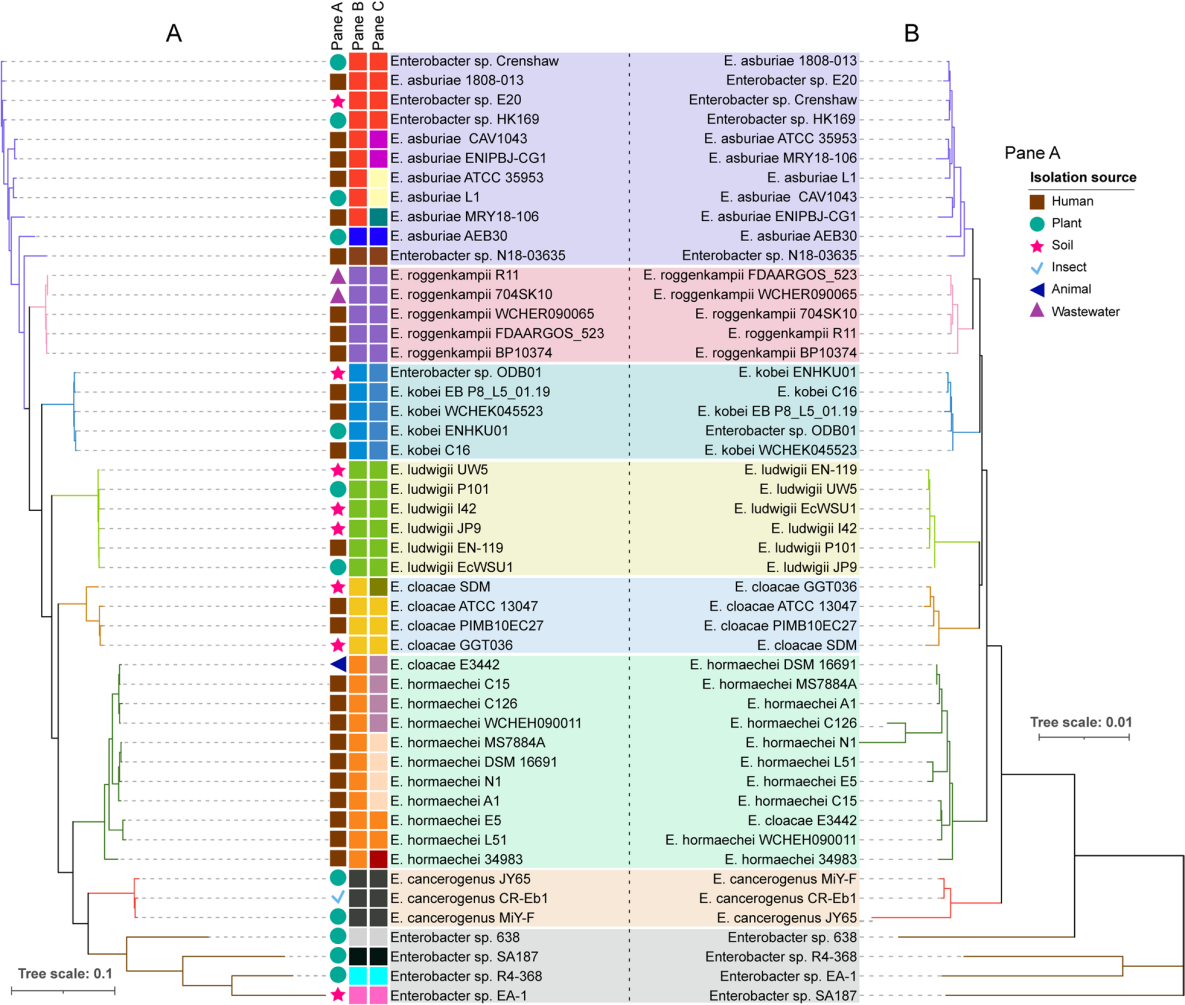
Phylogenetic analysis based on concatenated amino acid sequence of different genes among 49 *Enterobacter* strains. **A**, phylogenetic tree based on 46 core genes; **B**, phylogenetic tree based on 65 house-keeping genes. Pane A shows the isolation resource of each species. The color of pane B and pane C indicate species and subspecies according to their average amino acid identity (AAI) and average nucleotide identity (ANI) values (Additional file 1)

To confirm the updated classification of the 49 *Enterobacter* genomes, we employed phylogenetic analyses based on the concatenated amino acid sequences of core and house-keeping genes of the 49 *Enterobacter* genomes, of which the results showed that strains Crenshaw, E20 and HK169 were grouped with *E. asburiae* strains, while strain ODB01 was classified into the same clade containing *E. kobei* strains only (Fig. 1), consistent to the conclusions from AAI and ANI assays. Thus, our results suggested that genome sequence-based methodologies, such as AAI and ANI analyses, can be successfully applied for the taxonomic assignment of *Enterobacter* species.

### Core- and pan-genome analyses reveal high genetic diversity of *Enterobacter* species

The core genome is essential to the basic lifestyle of bacteria, while the pan genome provides species diversity, environmental adaptability and other characteristics [23]. Pan-genomics analysis provides an efficient method for studying and modeling genomic diversity, evolution, adaptability, and population structures [43].To further evaluate the genetic diversity in *Enterobacter* genus, we performed core- and pan-genome analyses of the 49 genomes. The genomes of the 49 strains have 16,201 pan genes, 46 core genes and 169 singleton genes (Additional file 2), indicating that *Enterobacter* has high genetic diversity. In addition, core- and pan-genome ratio (R_CP_=core-genome/pan-genome) were calculated for each genome. Higher the R_CP_ value indicates the smaller proportion of the mutated genes in the genomes. The genome of *E. hormaechei* had the lowest R_CP_ value (0.03) and *E. cancerogenus* had the highest R_CP_ value (0.75) (Additional file 2). These data demonstrated that *E. hormaechei* might be more genetically diversified than other *Enterobacter* species, while *E. cancerogenus* strains possessed more conserved genomes.

Core- and pan-genome ratio (R_CP_) can be used as an effective predictor for bacterial genetic diversity, but the value will change as increased number of genomes will change the size of pan-genome and core genome [44]. This is especially true for species with open pan-genomes, the size of which increases logarithmically as the number of genomes increased [45]. Compared with pan-genome analyses of other bacteria, such as *Propionibacterium acnes* (0.88) [46] and *Erwinia amylovora* (0.89) [47], the R_CP_ value of *Enterobacter* is relatively low (0.003-0.75), which may be due to all analyses being based on the species level (Additional file 2). However, despite their diverse lifestyles, *Enterobacter* genomes still have an important set of core functions that reflect their adaptability to the environment and the metabolic strategies they preserve for survival.

**Fig. 2:**
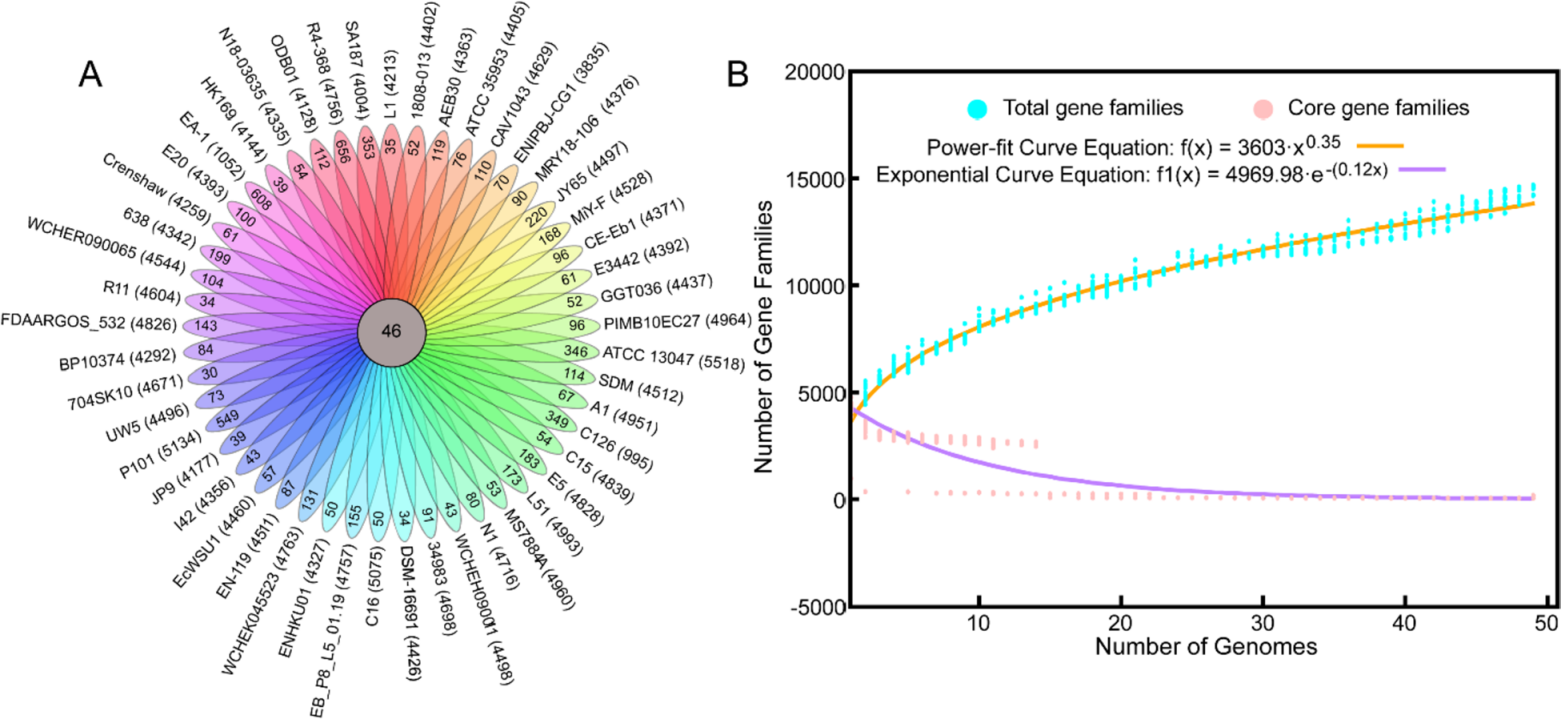
The Venn diagram and prediction of pan-genome in *Enterobacter* species. **A** Flower plots show the core gene number (in the center) and strain-specific gene number (in the petals) in *Enterobacter* strains. The numbers following the strain name represent the total number of coding proteins. **B** the pan- and core-genome size prediction using Bacterial Pan-Genome Analysis Pipeline (BPGA)

For the sake of evaluating the genetic discrepancies among the 49 *Enterobacter* strains, we further calculated the number of specific genes of each strain by clustering a total of 215,761 coding sequences (CDS) across the genomes. In total, 6,633 specific genes were identified (Fig. 2A). The number of specific genes harbored by each strain varied in a broad range from 35 (*E. asburiae* L1) to 656 (*Enterobacter* sp. R4-368) (Fig. 2A), consistent to the variant principal genome features of the 49 strains (Table 1). Besides, we drew the fitting curves using the power-law regression model based on Heap’s Law with a fitted exponent γ = 0.35 to predict the development trends of the pan-genomes of *Enterobacter* genus and different *Enterobacter* species (Fig. 2B). Based on the patterns of the curves, the pan-genomes of both *Enterobacter* genus and different *Enterobacter* species were open (Fig. 2B and Additional file 3). The open pan-genome result showed that 49 analyzed strains only represent a subset of the genetic diversity of *Enterobacter*, indicating that the extensive genome is still evolving through gene acquisition and diversification. This result is consistent with previous studies on *Enterococcus* [20] and *Enterobacter cloacae* [48], demonstrating that the genes in *Enterobacter* constantly exchange within and between bacterial species. Although some features may be absorbed and integrated into the genome, only those features that have survival benefits to the organism will remain, while the rest will be discarded by highly active rearrangement events [20].

**Fig. 3:**
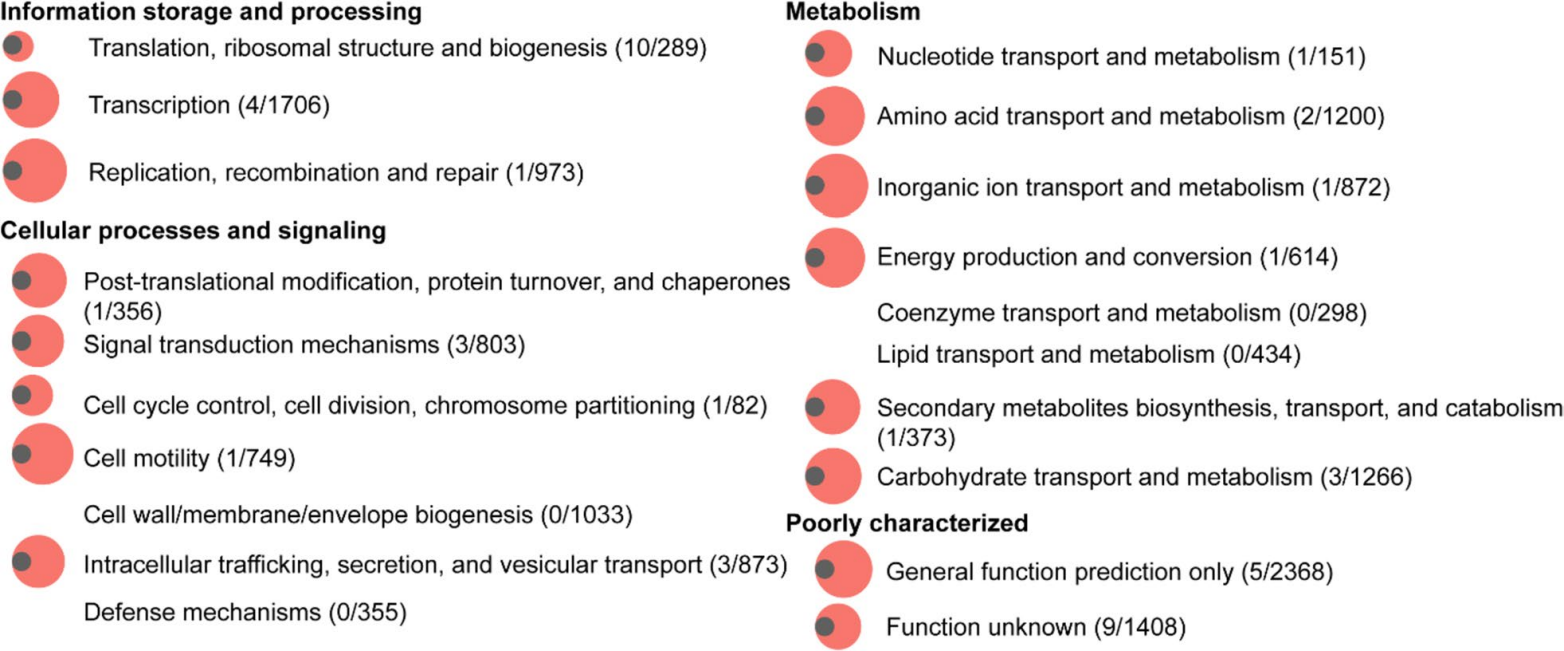
Expansion of the pan-genome compared with the core-genome for each functional category in *Enterobacter* species. All data in Clusters of Orthologous Groups (COG) was normalized by min-max normalization. The number of core genes in each functional category was normalized (grey circle). The magnification of the pan gene number compared with that of core gene in each functional category is shown by the red circle. The COG categories with no circles contains only pan genes

In order to find the differences of gene functions among *Enterobacter* species, gene annotation using the Clusters of Orthologous Groups (COG) database were determined and compared. Our results showed that the pan-genome expanded in most categories with varying degrees compared to the core genome (Fig. 3). The largest expansion was detected in the group “replication, recombination and repair” with 1 ratio of 973. Functions of genes in this group are related to host-environment interactions, and may also be involved in niche adaptation, possibly acquired by mobile genetic elements [49]. In the categories “cell motility” and “inorganic ion transport and metabolism”, the pan gene numbers are 749 and 872 times more than that of core genes, respectively. Moreover, all genes involved in “cell wall/membrane/envelope biogenesis”, “defense mechanisms”, “coenzyme transport and metabolism”, “lipid transport and metabolism” are pan genes, suggesting these genes may contribute to their adaptation to diverse hosts or environmental conditions. In summary, the data obtained from the core- and pan-genome analyses revealed the high genetic diversity in *Enterobacter* species.

### Differences of secretion system gene clusters among *Enterobacter* strains

**Fig. 4:**
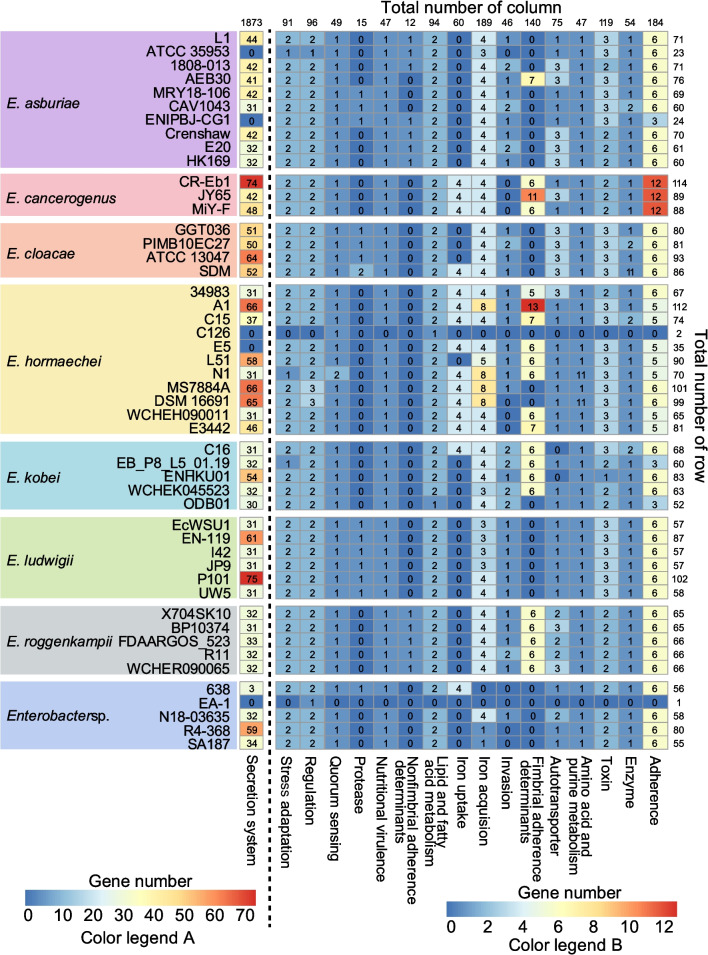
Heatmap analysis of virulence factors in *Enterobacter* species. Heatmap analysis shows the number and category of virulence factor in each strain. The color in secretion system based on the color legend A, while the color in all other virulence factors based the color legend B

A total of 3,391 potential virulence factors were detected in the 49 *Enterobacter* strains, averaging 68 per genome (Fig. 4). These virulence factors were mainly involved in secretion system (1,873), iron acquisition (189), adhesion (184), fimbrial adherence determinants (140) and toxin (119). Protein secretion systems were the largest component of virulence factors, which are critical for both pathogenic and nonpathogenic organisms to exploit nutrient resources within a host or specific niche and to evade recognition by the immune system [50]. Interestingly, the distribution of the virulence factors exhibited obvious species-specific patterns. For example, protease mainly existed in *E. ludwigii* and *E. cloacae*, while nonfimbrial adherence determinants were mainly found in *E. ludwigii*., iron uptake system was primarily detected in *E. cancerogenus* and *E. hormaechei*, while fimbrial adherence determinants mainly existed in *E. roggenkampii*, *E. hormaechei*, *E. kobei* and *E. cancerogenus*.

**Fig. 5:**
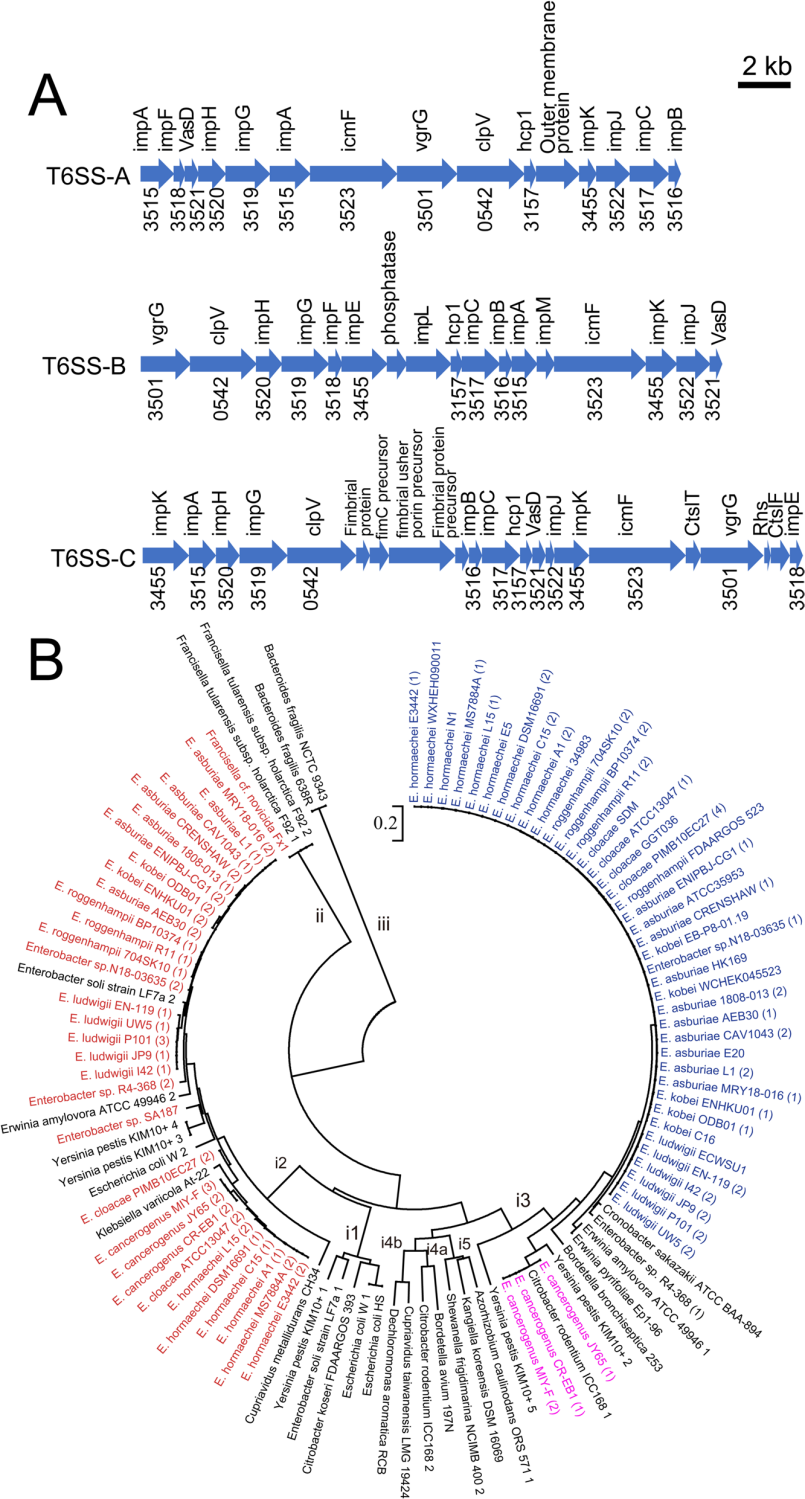
Gene organization and phylogenetic relationships of type VI secretion system (T6SS) gene clusters in *Enterobacter* species. **A** Schematic representation of the identified T6SS genes in *Enterobacter* species. Three types of T6SS gene clusters were detected among the 49 strains, named T6SS-A, T6SS-B and T6SS-C. The Clusters of Orthologous Groups (COG) IDs were presented under the genes in the clusters. **B** The phylogenetic trees was constructed using amino acid sequences of TssC in *Enterobacter* strains and 31 well-studied T6SSs (black). In general, T6SS gene clusters were classified into 3 groups (i-iii), and the group i was composed of six subfamilies (i1, i2, i3, i4a, i4b and i5). Our *Enterobacter* T6SSs were shown in red, pink and blue colors

The secretion of many important bacteria virulence factors is related to the secretion systems, including type VI secretion system (T6SS) [51]. T6SS participates in the pathogenicity of bacteria and is related to the formation of biofilm, recognition of competitor and stress responses and bacterial resistance acquisition [52, 53]. Based on the core components, we discovered three distinct types of T6SS gene clusters among the 49 strains, which were designated as T6SS-A, T6SS-B and T6SS-C (Fig. 5A). Additionally, based on the amino acid sequence of TssC protein, T6SS-A belonged to subtype i2, while T6SS-B and T6SS-C belonged to subtype i3 (Fig. 5B). Although T6SS-C is phylogenetically close to T6SS-B, no similarity in the core gene organization was found between T6SS-C and T6SS-B. The phylogenetic tree suggested that the different types of T6SS in *Enterobacter* were acquired by independent horizontal gene transfer [54].

**Fig. 6:**
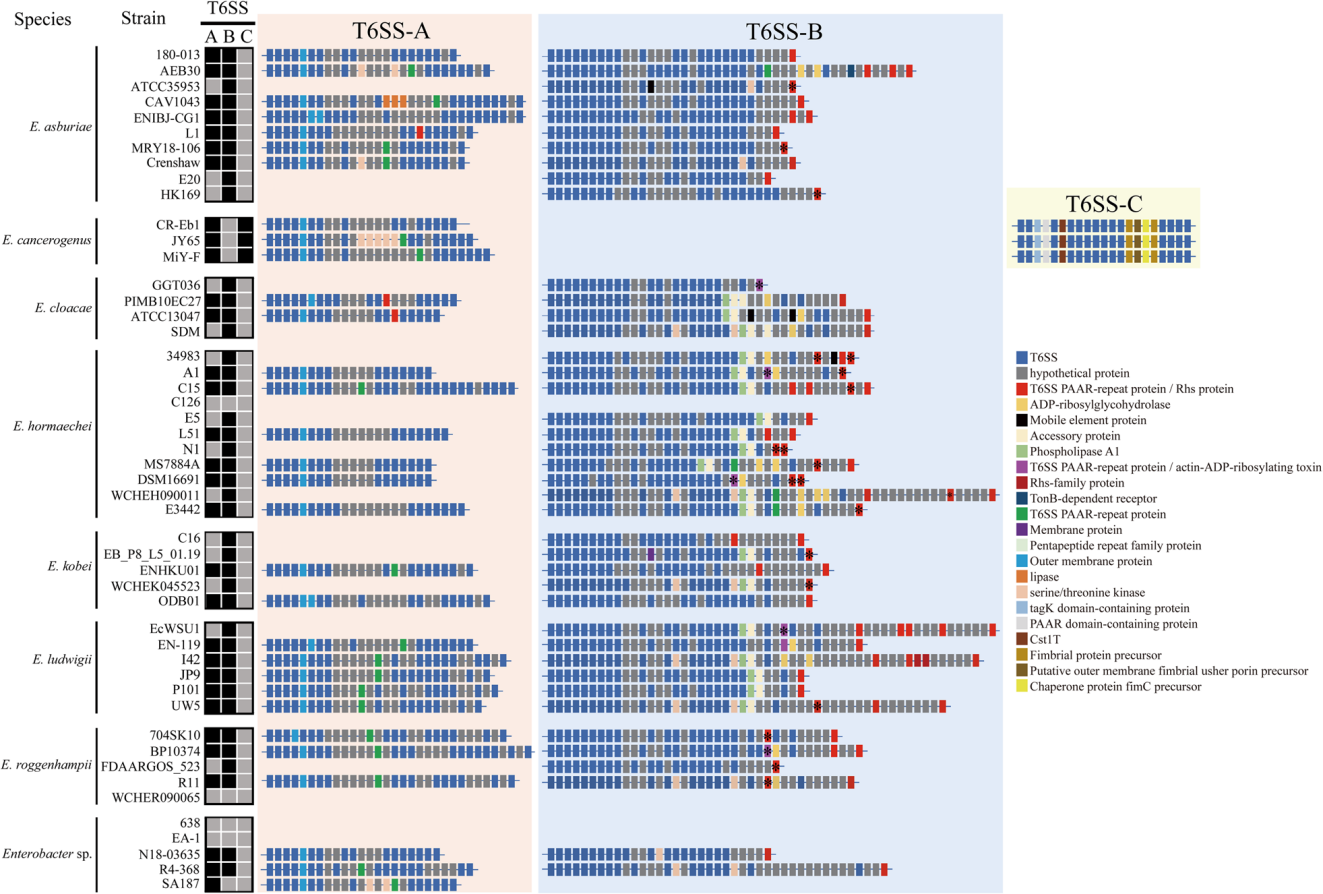
Gene organization of T6SS gene cluster in *Enterobacte*r species. Heatmap shows the presence (black) or absence (grey) of T6SS gene cluster. T6SS cluster in composed of conseved T6SS core component genes (blue boxes) and variable genes. In each type, they have their own specific gene, such as outer membrane protein in T6SS-A, PAAR repeat protein with Rhs repeat (red color or purple color) or C-terminal toxin domain (red color or purple color with asterisk) in T6SS-B, chaperone-usher fimbrial biogenesis proteins in T6SS-C. T6SS: type VI secretion system

**Fig. 7:**
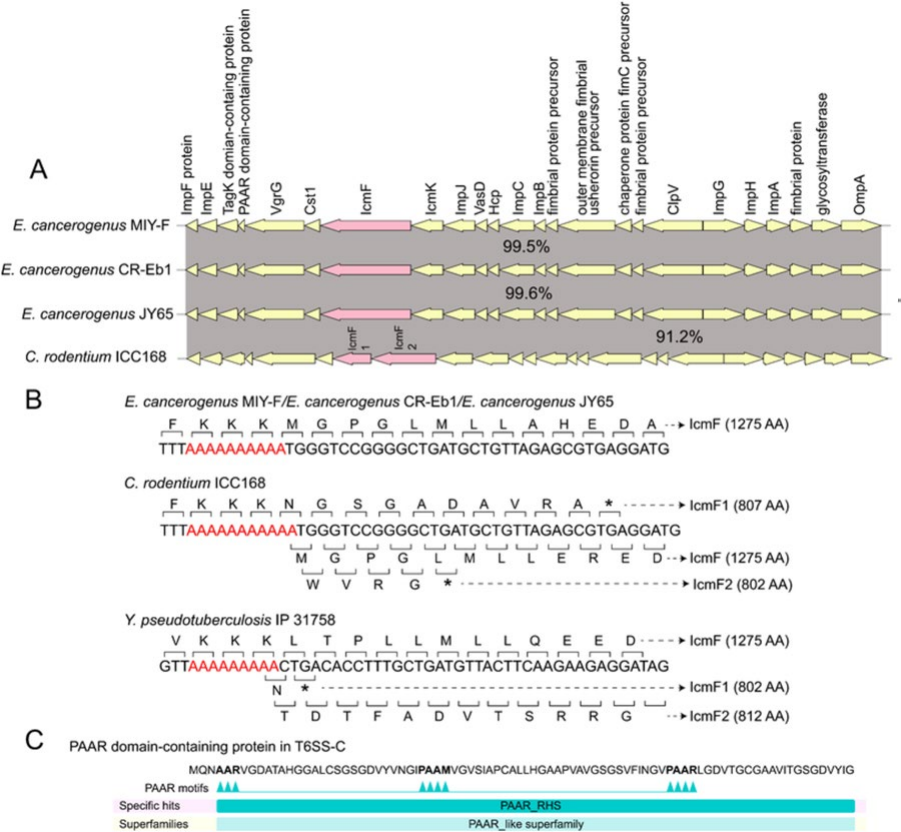
The *icmF* gene encodes frameshift mutations in different species. **A** the gene structure comparison of T6SS-C in three *Enterobacter cancerogenus* strains and T6SS gene cluster in *Citrobacter rodentium* ICC168. The percentage means the amino acid sequence identity. **B** Representation of the nucleotide sequences of five strains encoding icmF protein. The poly-A tract is shown in red. The codons and the translation result are shown, as well as those mutations produced from frameshifting are indicated (stop codon marked by the asterisks) with their corresponding size. **C** Prediction of the conserved domain of the PAAR domain-containing protein in T6SS-C

Different numbers of secretion systems were identified in 49 strains. For example, no T6SS was detected in three strains including *E. hormaechei* C126, *Enterobacter* sp. 638, and *Enterobacter* sp. EA-1, one type of T6SS was found in 15 strains, and two types of T6SS were found in 31 strains. More than 93% of the strains harbored at least one functional T6SS and conserved in specific species, showing that T6SS was probably acquired from the common origin before the diversification of species within the genus [14]. The diverse *Enterobacter* T6SSs indicates the effect of selection pressures during the bacterial evolution and adaptation to the environments [55]. Interestingly, not only multiple T6SSs were present in one strain, but also the T6SS gene structures were different (Fig. 6), suggesting that these T6SS were not originated from duplication but by independent acquisition [37]. B Patricia, PA Luke, F Alain and AL Maria [56] suggested that several T6SS clusters in *Pseudomonas putida* were the result of duplication. However, *Enterobacter* strains with more than one T6SS normally contain clusters from different subfamilies or different gene structures within the same subfamily (Fig. 6), demonstrating the probability of horizontal gene transfer of T6SS [14, 54]. In addition, although multiple T6SSs are commonly found in most strains, no identical T6SSs in one or cross strains are found, demonstrating that different types of T6SSs involve in different biological function [55]. This differentiation indicates the different origins of T6SS and differences in functions and structures [57]. T6SS-A gene cluster was detected in 31 strains, T6SS-B was observed in 41 strains, suggesting its dominant presence. T6SS-C was restricted to *E. cancerogenus* strains (Fig. 6) with highly conserved gene cluster (>99% amino acid similarity), exhibiting significant species-specificity (Fig. 7A). Additionally, T6SS gene cluster in *C. rodentium* is almost identical to T6SS-C in *E. cancerogenus* (91.2% amino acid similarity) (Fig. 7A).

Gene structures of three types of T6SS were significantly different and therefore T6SS could be used as markers to distinguish strains (Fig. 6). For example, outer membrane protein existed in all T6SS-A gene clusters. One or more T6SS PAAR-repeat proteins were found in T6SS-B (a total of 77 proteins) (Additional file 4), which could be used as a marker to separate with other two T6SSs. Among these 77 proteins, only 26 proteins have an N-terminal PAAR motif and a C-terminal toxin domain, while others with an N-terminal PAAR motif and a RHS repeat (Additional file 4). We found an interesting element, a transcriptional slippery site (poly-A tract), in T6SS-C *icmF* genes (Fig. 7B), which might produce different protein mutations. However, this element was not detected in other two T6SS *icmF* genes. In T6SS-C, we also found chaperone-usher fimbrial biogenesis proteins (fimbrial protein, chaperone protein and outer membrane fimbrial usher porin), which were not found in other types. In addition, BLAST comparison of PAAR domain-containing protein gene in T6SS-C showed that this gene was only found in *E. cancerogenus* (MiY-F, CR-Eb1 and JY65) and *C. rodentium* (DBS100, ICC168) (Additional file 5), suggesting that T6SS-C was probably acquired from a common ancestor *via* an event of independent horizontal gene transfer. The presence of chaperone-usher fimbria proteins in T6SS-C was only reported in *C. rodentium* T6SS gene cluster, which might be involved in gastrointestinal colonization [58]. A poly-A tract was identified in the *icmF* gene of *E. cancerogenus* T6SS-C (Fig. 7B). The *icmF* (*TssM*) gene in *C. rodentium* and *Yersinia pseudotuberculosis* with the transcriptional slippery site (poly-A tract) (Fig. 7B) could lead to frameshifting and product two *icmF* variants and a full-length canonical protein [59], suggesting that these genes could produce three different proteins as transcriptional frameshifting in *E. cancerogenus*, which needs further experimental verification.

Rearrangement hotspot proteins (Rhs) proteins also played a primary determinant in intercellular competition [15]. *Dickeya dadantii* 3937 *Rhs* genes carried with C-terminal toxin domains deployed its function in intercellular inhibition [60].Further protein structure analysis of IcmF revealed a PAAR motif but different from the classical Rhs and without C-terminal toxin domain (Fig. 7C), suggesting that IcmF is a truncated protein lack of effective interspecies competition [60]. We also found four types of PAAR-domain containing proteins, and some of them are fused to Rhs proteins [61]. Here, we also found four types of this protein, including T6SS PAAR-repeat protein/Rhs protein, T6SS PAAR-repeat protein/actin-ADP-ribosylating toxin, T6SS PAAR-repeat protein and PAAR domain-containing protein (Fig. 6). J Ma, M Sun, W Dong, Z Pan, C Lu and H Yao [62] demonstrated that Rhs proteins with an N-terminal PAAR motif and a C-terminal toxin domain could promote colonization and fitness during infection.

## Conclusion

In summary, analysis of 49 *Enterobacter* genomes demonstrated that their core- and pan-genome are open, and a number of virulence factors were characterized. Comparative genomic analysis revealed that the secretion systems are the largest among virulence factors, involved in the pathogenicity and niche adaptation. T6SS gene clusters were detected in most *Enterobacter* strains. The composition of the T6SS gene clusters varied, but its core components were found to be highly conserved. A total of three different types of T6SS were detected by comparative genomics. T6SS-A, and T6SS-B were widely distributed in *Enterobacter* species. A novel type of T6SS, named T6SS-C, was only detected in in *E. cancerogenus*. Whilst this type of T6SS gene cluster is not available in other *Enterobacter* species, the same type of T6SS was detected in the remotely related genus *Citrobacter*, suggesting that T6SS was probably acquired by an event of horizontal gene transfer. It is likely that the different types of T6SSs are involved in different biological functions and contribute to the evolutional adaptation.

### Supplementary Information

**Additional file 1.** Similarity identity matrix of average amino acid identity (AAI) (upper triangle) and average nucleotide identity (ANI) (lower triangle) among *Enterobacter* species. The color represents the numerical size, red color means the highest value, green color represents the lowest value.Strains are considered one species when share >95% AAI and ANI.

**Additional file 2.** Pan-genome features in different *Enterobacter* species.

**Additional file 3.** Pan-genome, core-genome and singleton development of different *Enterobacter* species.

**Additional file 4.** Prediction of conserved domain in T6SS PAAR-repeat proteins (only list the protein with N-terminal PAAR motif and a C-terminal toxin domain).

**Additional file 5.** BLAST comparison and taxonomic analysis based on PAAR domain-containing protein in T6SS-C gene cluster. The phylogenetic tree is inferred using the Neighbor-Joining method.

## Data Availability

All data generated or analysed during this study are included in this published article and its supplementary information files.
