# Supplementary material for: High genetic diversity and different type VI secretion systems in Enterobacter species revealed by comparative genomics analysis
Source: BMC Microbiol. 2024 Jan 19;24:26. doi: 10.1186/s12866-023-03164-6 (PMC10797944; doi:10.1186/s12866-023-03164-6)
Supplement: Supplementary file 1 — Additional file 1. Similarity identity matrix of average amino acid identity (AAI) (upper triangle) and average nucleotide identity (ANI) (lower triangle) among Enterobacter species. The color represents the numerical size, red color means the highest value, green color represents the lowest value.Strains are considered one species when share >95% AAI and ANI. [file 12866_2023_3164_MOESM1_ESM.docx]

**Additional file 1** Similarity identity matrix of average amino acid identity (AAI) (upper triangle) and average nucleotide identity (ANI) (lower triangle) among *Enterobacter* species. The color represents the numerical size, red color means the highest value, green color represents the lowest value. Strains are considered one species when share >95% AAI and ANI.
