# Supplementary material for: High genetic diversity and different type VI secretion systems in Enterobacter species revealed by comparative genomics analysis
Source: BMC Microbiol. 2024 Jan 19;24:26. doi: 10.1186/s12866-023-03164-6 (PMC10797944; doi:10.1186/s12866-023-03164-6)
Supplement: Supplementary file 2 — Additional file 2. Pan-genome features in different Enterobacter species. [file 12866_2023_3164_MOESM2_ESM.docx]

**Additional file 2** Pan-genome features in different *Enterobacter* species

| Species | Genome number | Core-genes | Pan-genes | Singleton genes | R_CP_* |
| --- | --- | --- | --- | --- | --- |
| *E. asburiae* | 11 | 2944 | 6798 | 61 | 0.43 |
| *E. cancerogenus* | 3 | 3905 | 5193 | 229 | 0.75 |
| *E. cloacae* | 5 | 3762 | 6239 | 126 | 0.60 |
| *E. hormaechei* | 10 | 217 | 7379 | 98 | 0.03 |
| *E. kobei* | 5 | 3362 | 5753 | 202 | 0.58 |
| *E. ludwigii* | 6 | 3842 | 6176 | 61 | 0.62 |
| *E. roggenkampii* | 5 | 3544 | 5829 | 100 | 0.61 |
| *Enterobacter* sp*.* | 4 | 328 | 6767 | 541 | 0.05 |
| All | 49 | 46 | 16201 | 169 | 0.003 |

*Rcp: Core-genome: pan-genome ratio
