# Supplementary material for: High genetic diversity and different type VI secretion systems in Enterobacter species revealed by comparative genomics analysis
Source: BMC Microbiol. 2024 Jan 19;24:26. doi: 10.1186/s12866-023-03164-6 (PMC10797944; doi:10.1186/s12866-023-03164-6)
Supplement: Supplementary file 3 — Additional file 3. Pan-genome, core-genome and singleton development of different Enterobacter species. [file 12866_2023_3164_MOESM3_ESM.docx]

**Additional file 3** Pan-genome, core-genome and singleton development of different *Enterobacter* species

| Species | Pan-genome development  n=k*N^γ^ | Core-genome development  F(C)=K_C_*exp(-x/C) + Ω | Singletons development  F(S)=K_S_*exp(-x/S) + tg(θ) |
| --- | --- | --- | --- |
| *E. asburiae* | 4029.750*N^0.216^ | 1590.218*exp(-x/9.452) + 2450.211 | 594.169*exp(-x/2.473) + 126.896 |
| *E. cancerogenus* | 4273.122*N^0.234^ | 1182.551*exp(-x/3.764) + 3107.343 | 1968.527*exp(-x/0.87) + 316.180 |
| *E. cloacae* | 446.661*N^0.236^ | 1372.683*exp(-x/2.615) + 3243.036 | 1947.084*exp(-x/1.018) + 307.531 |
| *E. hormaechei* | 4070.778*N^0.244^ | 4951.375*exp(-x/5.087) + 0.000 | 5759.976*exp(-x/0.875) + 175.010 |
| *E. kobei* | 4304.314*N^0.180^ | 1644.728*exp(-x/5.183) + 2734.116 | 1624.433*exp(-x/1.013) + 186.184 |
| *E. ludwigii* | 4274.890*N^0.178^ | 576.149*exp(-x/3.291) + 3786.518 | 653.471*exp(-x/1.272) + 200.468 |
| *E. roggenkampii* | 4353.598*N^0.178^ | 1110.607*exp(-x/2.382) + 3415.463 | 1442.592*exp(-x/1.165) + 194.323 |
| *Enterobacter* sp*.* | 3921.572*N^0.395^ | 6049.666*exp(-x/1.902) + 0.000 | 20774.380*exp(-x/0.635) + 515.188 |

n = expected number of genes

N = number of genomes

‘k’ and ‘γ’ are proportionality constant and exponent, respectively, estimated by using the nonlinear least-squares curve fit to the mean values

F(C) = expected core-genome size

x = number of genomes, K_C_, C and Ω are constants required for fitness.

F (S) = expected singleton number

K_S_, S and tg(θ) are constants
