# Supplementary material for: High genetic diversity and different type VI secretion systems in Enterobacter species revealed by comparative genomics analysis
Source: BMC Microbiol. 2024 Jan 19;24:26. doi: 10.1186/s12866-023-03164-6 (PMC10797944; doi:10.1186/s12866-023-03164-6)
Supplement: Supplementary file 5 — Additional file 5. BLAST comparison and taxonomic analysis based on PAAR domain-containing protein in T6SS-C gene cluster. The phylogenetic tree is inferred using the Neighbor-Joining method. [file 12866_2023_3164_MOESM5_ESM.doc]

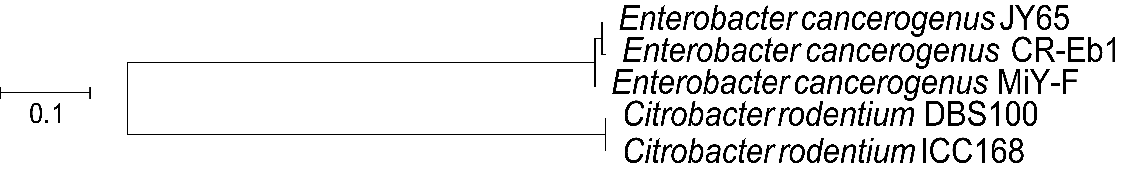


**Additional file 5** BLAST comparison and taxonomic analysis based on PAAR domain-containing protein in T6SS-C gene cluster. The phylogenetic tree is inferred using the Neighbor-Joining method.
